# Hepatic PKA inhibition accelerates the lipid accumulation in liver

**DOI:** 10.1186/s12986-019-0400-5

**Published:** 2019-10-11

**Authors:** Jining Yang, Xiaoying Zhang, Long Yi, Ling Yang, Wei Eric Wang, Chunyu Zeng, Mantian Mi, Xiongwen Chen

**Affiliations:** 1 Research Center for Nutrition and Food Safety, Chongqing Key Laboratory of Nutrition and Food Safety, Institute of Military Preventive Medicine, Third Military Medical University, Chongqing, People’s Republic of China; 2 Department of Physiology & Cardiovascular Research Center, Temple University Lewis Katz School of Medicine, Philadelphia, PA USA; 3 Department of Medical Genetics & Molecular Biochemistry, Temple University Lewis Katz School of Medicine, Room 624 Kresge Hall, Philadelphia, PA USA; 4 Daping Hospital, The Third Military Medical University, Chongqing, People’s Republic of China

**Keywords:** Protein kinase a, Non-alcoholic fatty liver diseases, Lipid metabolism, RNA sequencing

## Abstract

**Background/aims:**

Liver lipid accumulation induced by high-fat diet (HFD) is an early onset process of non-alcoholic fatty liver diseases (NAFLD). Protein kinase A (PKA) is known to be involved in hepatic lipid metabolism. However, the role of PKA in NAFLD has not been well tested in vivo due to the lack of optimal PKA deficient mouse model.

**Methods:**

A novel PKA-specific inhibitor gene was conditionally overexpressed in mouse (PKAi mouse) liver using LoxP/Cre system. PKA activity in the liver extract was measured with a commercial assay kit. The PKAi and control mice of 8-week age, were subjected to HFD or chow diet (CD) for 2 months. Body weight, liver index, and triglyceride in the liver were measured. RNA sequencing was performed for the liver tissues and analyzed with Gene Ontology (GO) and pathway enrichment.

**Results:**

PKAi-GFP protein was overexpressed in the liver and the PKA activation was significantly inhibited in the liver of PKAi mouse. When fed with CD, RNA sequencing revealed 56 up-regulated and 51 down-regulated genes in PKAi mice compared with control mice, which were mainly involved in lipid metabolism though no significant differences in the body weight, liver index, triglyceride accumulation were observed between PKAi and control mice. However, when fed with HFD for 2 months, the liver was enlarged more, and the accumulation of triglyceride in the liver was more severe in PKAi mice. When comparing the transcriptomes of CD-fed and HFD-fed control mice, GO enrichment showed that the genes down-regulated by HFD were mainly enriched in immune-related GO terms, and up-regulated genes were enriched in metabolism. When comparing the transcriptomes of CD-fed and HFD-fed PKAi mice, GO analysis showed that the down-regulated genes were enriched in metabolism, while the up-regulated genes were clustered in ER stress-related pathways. When comparing HFD-fed PKAi and HFD-fed control mice, the genes with lower expression level in PKAi mice were enriched in the lipoprotein synthesis, which might explain that more TG is accumulated in PKAi liver after HFD feeding.

**Conclusions:**

Reduced PKA activity could be a factor promoting the TG accumulation in the liver and the development of NAFLD.

## Background

Nonalcoholic fatty liver disease (NAFLD) consists a wide pathological spectrum ranging from steatosis, nonalcoholic steatohepatitis (NASH), fibrosis, and cirrhosis to hepatocellular carcinoma; and has become the fastest growing liver disease worldwide with a prevalence as high as a 25% of the population. NAFLD threatens the public health and causes a huge economic burden [1, 2]. Though several studies have detected molecules as potential therapeutic targets, there is no US FDA-approved therapy for NAFLD [2]. Therefore, it is necessary to understand the underlying mechanism of this disease to meet the need for drug discovery and treatment for patient benefit.

The underlying mechanism for the development and progression of NAFLD is complex and multifactorial, among which lipid accumulation in liver is an early step [3, 4]. There are multiple mechanisms leading to lipid accumulation in hepatocytes, including increased free fatty acids supply and de novo hepatic lipogenesis; and decreased free fatty β-oxidation in the liver as well as decreased hepatic lipid export [5]. Lipid accumulation may also result in the early “inflammatory” hit, thus leading to the whole spectrum of NAFLD pathologies [3].

Protein kinase A (PKA) plays a crucial role in the regulation of lipid metabolism. The dysregulation of PKA signaling is associated with the pathogenic mechanisms underlying many metabolic diseases, including NAFLD [6, 7]. The pharmacological studies show that the activity of PKA is linked to the pharmacological effects of the major lipid regulating agents [8–12]. For example, the well-known antioxidant resveratrol could reduce the lipid accumulation and alleviate NAFLD in a PKA-dependent way, suggesting that the activity of PKA might be a promising target in treating NAFLD [8]. However, the effect of activation of PKA in hepatic lipid synthesis seems to be conflicting. For example, the in vitro study showed that PKA activation by glucagon or forskolin downregulates the lipid synthesis efficiency in hepatocytes by phosphorylating cAMP response element binding protein (CREB) to increase the expression of ring finger protein-20 (RNF-20), which mediates the degradation of sterol regulatory element binding protein-1c (SREBP-1c) through ubiquitination [13]. On the other hand, PKA activation induced by thyrotropin in hepatocytes can upregulate SREBP-1c by phosphorylating peroxisome proliferators-activated receptors alpha (PPARα) to promote liver lipid synthesis [14]. Therefore, it is necessary to study the role of PKA in hepatocytes in the development of NAFLD in vivo and understanding the role of PKA in TG accumulation in the liver might provide promising strategies in developing therapy for NAFLD.

PKA is widely distributed in almost every type of cells of mammals, with a highly conserved structure among species [15]. The structure of PKA was first revealed in 1991 [16] as a heterotetramer consisting of 2 regulatory (PKA R) subunits that have 4 isotypes (RIα, RIIα, RIβ, RIIβ) and 2 catalytic subunits (PKA C) that have 4 isotypes (Cα, Cβ, Cγ, Prkx). The catalytic subunits are bound to the regulatory subunit dimer and stay inactive when cAMP level is low, because the regulatory subunit contains a pseudosubstrate domain to inhibit the kinase activity of the catalytic subunit [17]. When cytosolic cAMP is increased, the PKA R binds to cAMP, causing a conformational change that decreases its affinity for catalytic subunits and leads to the release of catalytic subunits, which then become active and phosphorylate serine and threonine in its target proteins. The activation of PKA plays a crucial role in lipolysis through many PKA targets involved in lipid metabolism, such as hormone sensitive lipase (HSL) [18]. Due to the existence of various isotypes of the subunits of PKA encoded by multiple genes in the same cell, it remains almost impossible to generate a hepatocyte-specific complete PKA knockout animal model to explore its roles in hepatic lipid metabolism in vivo. Genetically, to knockout all three PKA catalytic genes is very difficult. There is no PKA specific inhibitor neither [19] and PKA inhibition drug cannot achieve tissue specificity. For example, the widely used classical PKA inhibitor H89 can partially inhibit other proteins such as protein kinase B (PKB) [19]. Thus, it remains unclear whether hepatic PKA promotes or inhibits lipid accumulation in vivo. To circumvent this problem, we took advantage of the Cre/LoxP system [20] and constructed a transgenic mouse model that would overexpress a designed PKA inhibition (PKAi) fusion gene, containing the nucleotide sequence coding the amino acids 1–25 of PKA inhibitor peptide (PKI)-α and green fluorescent protein (PKAi-GFP) [21] specifically in the liver. PKAi contains the PKA inhibition domain of PKI-α, a naturally coded protein. PKAi, as a pseudosubstreate, interacts with the peptide/protein binding portion of the active site of the catalytic subunit in a competitive manner with a low nanomolar Ki value, thus inhibiting the activity of PKA [22].

After confirming the hepatic inhibition of PKA in the mouse model, we studied the body weight, liver index and triglyceride accumulation in the liver of mice fed with chow diet (CD) or high-fat diet (HFD). Also, unbiased RNA sequencing was done to elucidate the differences in transcription upon the inhibition of PKA in the liver of mice fed with normal or HFD chow. We found that PKA inhibition aggregates triglyceride accumulation in the liver after HFD feeding. Our findings provided new direct evidence regarding the role of PKA in lipid metabolism in vivo, and elucidated the mechanisms based on transcriptome analysis, which might open new avenues to combat NAFLD.

## Methods

### Experimental animals and treatments

**Fig. 1:**
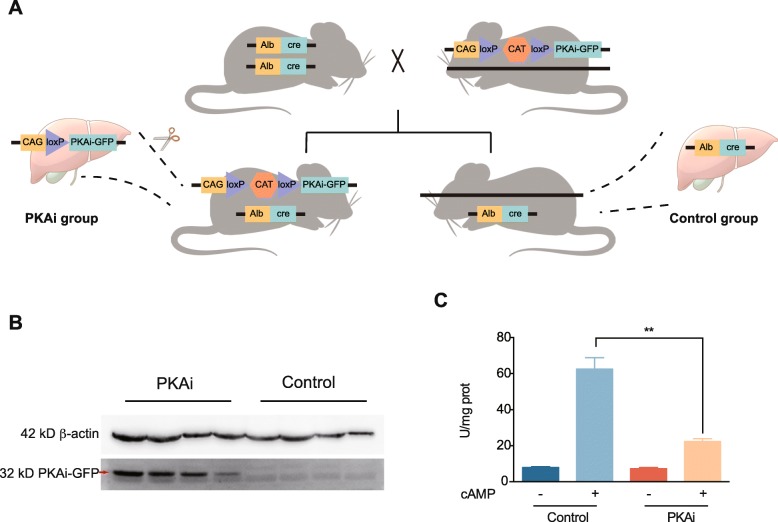
The hepatic-selective PKA-specific inhibition mouse models. **a**. The schematic illustration for the generation of hepatocyte-specific PKA inhibition animal models. **b**. Western blot for the detection of PKAi-GFP in the liver of PKAi and control mice. **c**. PKA activity stimulated by 1 μmol/L cAMP of the liver tissue of PKAi or control mice. **p < 0.01 vs control by two-way ANOVA with Bonferroni adjustment for mean comparison. (*N* = 8)

Establishment of PKAi-GFP transgenic mice: A DNA sequence coding a peptide (animo acids sequence: TDVETTYADFIASGRTGRRNAIHD) of the PKA inhibitory domain of mouse protein kinase inhibitor α (mouse Entrez gene ID 18767) was synthesized and subcloned into plasmid pAcGFP1-N1 (Clontech) to make a PKAi-GFP fusion gene. Then this fusion gene was subcloned into the CAG-LoxP-CAT-LoxP vector provided by Dr. Qinglin Yang (Louisianan State University). Then the transgenic mouse line carrying CAG-LoxP-CAT-LoxP-PKAi-GFP was made by the Institute of Model Animal of Wuhan University. In this transgenic mouse line, the expression of PKAi-GFP was blocked by a stop sequence (a bacteria chloramphenicol, acetyltransferase gene, CAT) [23]. This mouse line was crossbred with the hepatocyte-specific cre (Alb-cre) homozygous mouse line to obtain the double transgenic (CAG-LoxP-CAT-LoxP-PKAi-GFP/Alb-cre) mice. In hepatocytes of the double transgenic mice, CAT was excised by cre, and then PKAi-GFP expression was induced. Littermate mice with only a single transgene (Alb-cre or CAG-LoxP-CAT-LoxP-PKAi-GFP) and with matched age and sex were used as controls. (Fig. 1a).

Treatments: male PKAi mice (*n* = 12) and male control mice (n = 12) were fed with CD till 8-week old, and then divided randomly into four groups (6 mice/group): PKAi mice fed with CD (PKAi_CD), PKAi mice fed with HFD (PKAi_HFD), control mice fed with CD (control_CD) and control mice fed with HFD (control_HFD). Mice were then fed with either CD (10% calories from fat, D12350B, Research Diets, US) or HFD (45% calories from fat, D12451, Research Diets, US) for 2 months. Detailed information of the diets was displayed in Additional file 1. All animals were maintained under a regular 12-h light/12-h dark period at a controlled temperature (22 ± 2 °C) and humidity (50 ± 5%), and received food and water ad libitum. Body weight and diet consumption were recorded weekly during the study. Liver samples of the same region of left lobe of each mice were collected for the following experiments. Animal care and treatments were conducted according to established guidelines and protocols approved by the Animal Care and Use Committee of the Third Military Medical University.

### Western blot

Whole protein samples extracted by T-PER lysis buffer (Thermo Fisher Scientific Inc., US) from liver tissues of 4 PKAi and 4 control mice (male, 8-week old) were used to determine the abundance of PKAi-GFP with anti-GFP antibody (Abcam, USA) by standard Western blotting protocol. β-actin (Cell Signaling Technology, USA) was used as the loading control. Briefly, equal amounts (20 μg) of proteins were subjected to 10% sodium dodecyl sulfate polyacrylamide gel electrophoresis and then electroblotted onto polyvinylidene difluoride membranes, which were subsequently blocked with TBST containing 5% BSA for 1 h before probed with primary antibodies overnight at 4 °C. Membranes were then incubated with a secondary anti-rabbit antibody (Cell Signaling Technology, USA), and the immunostained bands were visualized with Immobilon Western Chemiluminescent HRP Substrate (Millipore, USA) and captured by a Fusion FX (Vilber Lourmat, France) machine. The antibody information was displayed in Additional file 2.

### RNA isolation and quantitative real-time polymerase chain reaction (qRT-PCR)

To determine PKAi-fusion gene expression, various tissues of the PKAi mice (8-week old) were harvested in RNAiso Plus reagent (Takara Bio, Japan), and the total RNA was extracted according to the manufacturer’s instructions. To prevent the genomic DNA contamination, we treated the RNA sample with TURBO DNA-free kit (Invitrogen, US). Then the RNA concentration and purity were measured by NanoDrop 2000 spectrophotometer (Thermo Fisher Scientific Inc., US). The first-strand cDNA was synthesized by a PrimeScript RT Master Mix kit (Takara Bio, Japan). qRT-PCR was carried out with the qTower 2.2 real-time PCR system (Anakytik Jena, Germany) using SYBR Premix Ex Taq II (Takara Bio, Japan). The primers for the targeted genes were synthesized by Sangon Biotech (Shanghai, China). The primer sequences used for gene expression analysis are listed in Additional file 3. The amplification profile consisted of denaturation at 95 °C for 30 s, followed by 40 cycles of 95 °C for 5 s and 60 °C for 30 s. A melting curve stage was performed at the end of the amplification. Relative fold-changes in gene expression were analyzed by the 2^−ΔΔCt^ method and normalized to the internal control gene β-actin [24].

### Measurement of PKA activity

The control and PKAi mouse liver samples were homogenized in lysis buffer supplemented with 0.4 mmol/L 3-isobutyll-methylxanthine (IBMX, a phosphodiesterase inhibitor, Sigma-Aldrich, US). Basal PKA activity or maximal PKA activity stimulated with 1 μM cAMP in the homogenates were assessed with a PKA kinase activity assay kit (Assay Design, Ann Arbor, MI), following the provided protocol. Briefly, 40 μL of samples and fully active recombinant PKA standard at different concentrations were added into the wells of the plate. Next, 10 μL of the reconstituted ATP was added to each of the well followed by the incubation at 30 °C for 90 min. After carefully aspirating the reagents, the plate was washed 4 times with 300 μL wash buffer and tapped to dry on clean absorbent towel. For the antigen-antibody binding, 25 μL of the Rabbit Anti-Phospho PKA Substrate Antibody and 25 μL Goat anti-Rabbit IgG HRP Conjugate were added to each well. Then the plate was sealed and incubated at room temperature for 60 min with shaking. Afterwards, the wells were aspirated again and rinsed 4 times with wash buffer. After the final wash, 100 μL of the TMB substrate solution was added to each well and incubate the plate at room temperature for 30 min. Finally, 50 μL of the stop solution was added to each well and the optical density was read by a plate reader at 450 nm. The PKA activity was calculated from the linear standard curve using linear regression and normalized to the protein concentration.

### RNA extraction, library preparation and sequencing

Mice of the four groups (PKAi_CD, PKAi_HFD, Control_CD and Control_HFD) were raised as described above. Then, 3 mice of each group were sacrificed and their liver tissues were harvested for RNA isolation using the TRIzol (Invitrogen, US) as described in the instruction. RNA quality was determined by examining A260/A280 with a Nanodrop™ OneCspectrophotometer (Thermo Fisher Scientific Inc., US). RNA Integrity was confirmed by 1.5% agarose gel electrophoresis. Qualified RNAs were finally quantified by Qubit3.0 with Qubit™ RNA Broad Range Assay kit (Life Technologies, US) and used for stranded RNA sequencing library preparation using KC™ Stranded mRNA Library Prep Kit for Illumina® (Wuhan Seqhealth Inc., China). PCR products corresponding to 200–500 bp were enriched, quantified and finally sequenced on Hiseq X10 sequencer (Illumina, US).

### RNA-seq data analysis

Sequencing reads that were in low quality or contained only adapters were pre-filtered and then the rest sequences were mapped to the mm9 genome using HISAT [25]. Clean reads were mapped to reference by featureCounts [26], and then reads per kilobase per million mapped reads (RPKMs) were calculated. We detected differentially expressed genes (DEGs) between groups with edgeR package [27]. Genes were called differentially expressed if they exhibited an adjusted *P* value< 0.05. Heatmap of the DEGs were generated using the heatmap R package [28]. Gene ontology (GO) enrichment analysis of DEGs were analyzed by KOBAS software [29] and the top 10 terms (ranked by q-value, the modified Fisher exact *P*-value) were displayed by the R package ggplot2 [30]. Gene set enrichment analysis (GSEA) [31] was used to investigate potential mechanisms in the Molecular Signatures Database (MSigDB) [32] of h (h.all.v6.2. symbols). The enrichment gene sets in GSEA with the lowest false discovery rate (FDR) was demonstrated. Metascape online tool [33] was used to do the network analysis. Briefly, DEGs of each group that were just up/down-regulated in control mice or just up/down-regulated in PKAi mice induced by HFD were put into two lists and uploaded to the website. Subsequently, enrichment analysis was performed online using Reactome Gene Sets [34] and Kyoto Encyclopedia of Genes and Genomes (KEGG) [35] database. Pathways with minimum overlap at 3, *P*-value cutoff at 0.01, and minimum enrichment at 1.5 were screened out and displayed in clusters. The thickness of lines between nodes represents the kappa score of overlapping genes of the linking nodes. The RNA-seq data entitled “Transcriptome studies of liver tissue in PKAi mouse model” were deposited to the Sequence Read Archive (SRA) with BioProject number PRJNA553077.

### Triglyceride assay

The triglyceride level of liver samples and the intracellular triglyceride content was determined with an enzymatic assay kit (Applygen Technologies Inc., Beijing, China) as described in the kit instruction. The protein concentrations were determined with a BCA Protein Assay Kit (Beyotime, China) as described previously. The results were expressed as mmol per gram protein (mmol/g prot).

### Hematoxylin & eosin (H&E) staining

Liver tissue was fixed in 4% paraformaldehyde, dehydrated, embedded in paraffin wax, and then sectioned into 5 μm-thick slices. The sections were subsequently deparaffined and rehydrated via a xylene and alcohol series before performing H&E staining according to standard histology procedure as previously described [36].

### Oil red O staining

Oil Red O powder (0.5 g, Sigma-Aldrich, USA) was evenly dissolved in 100 mL of isopropanol to prepare stock solution, which was then diluted with distilled water at the ratio of 3:2 and filtered to make the working solution. Liver tissue cryosections (8 μm) were prepared in a cryostat (Leica, Germany) and stained with prewarmed Oil Red O working solution for 15 min. After rinsing with distilled water three times, the sections were then counterstained with hematoxylin (Sigma-Aldrich, USA) for 3 min and gently washed with distilled water. Representative images were captured using a light microscopy (Leica, Germany).

### Statistical analysis

Data were reported as mean ± SEM and analyzed with SPSS 19.0 software (Chicago, USA). Statistical differences among groups were determined with Student’s t-test or two-way ANOVA. *P* values less than 0.05 were considered statistically significant.

## Results

### PKAi-GFP is specifically expressed in the mouse liver to suppress PKA activity

Hepatic-specific PKA inhibition mice and corresponding control mice were obtained by crossing the heterozygotes CAG-LoxP-CAT-LoxP-PKAi-GFP transgenic and Alb-cre transgenic mice (Fig. 1a). PKAi-GFP was expressed only in CAG-LoxP-CAT-LoxP-PKAi-GFP/Alb-cre double transgenic mice (termed PKAi mice here) but not in control mice, validated by western blots in mouse liver tissue (Fig. 1b). Tissue specific expression of PKAi-GFP was determined using qRT-PCR, showing that overexpressed PKAi-GFP was predominantly expressed in the liver but little in other tissues (Additional file 4). There was little PKA activity at baseline in both control and PKAi mice liver. However, when crude liver tissue extract was stimulated by 1 μmol/L cAMP, PKA activity was significantly increased in control mouse liver samples but not as much in PKAi transgenic samples, indicating that overexpressed PKAi-GFP in PKAi mouse liver was sufficient to inhibit most of cAMP-induced PKA activity (Fig. 1c).

### Transcriptome analysis of liver from PKAi and control mice showed fatty acid metabolism pathway being dominantly affected by hepatocyte-specific PKA inhibition

**Fig. 2:**
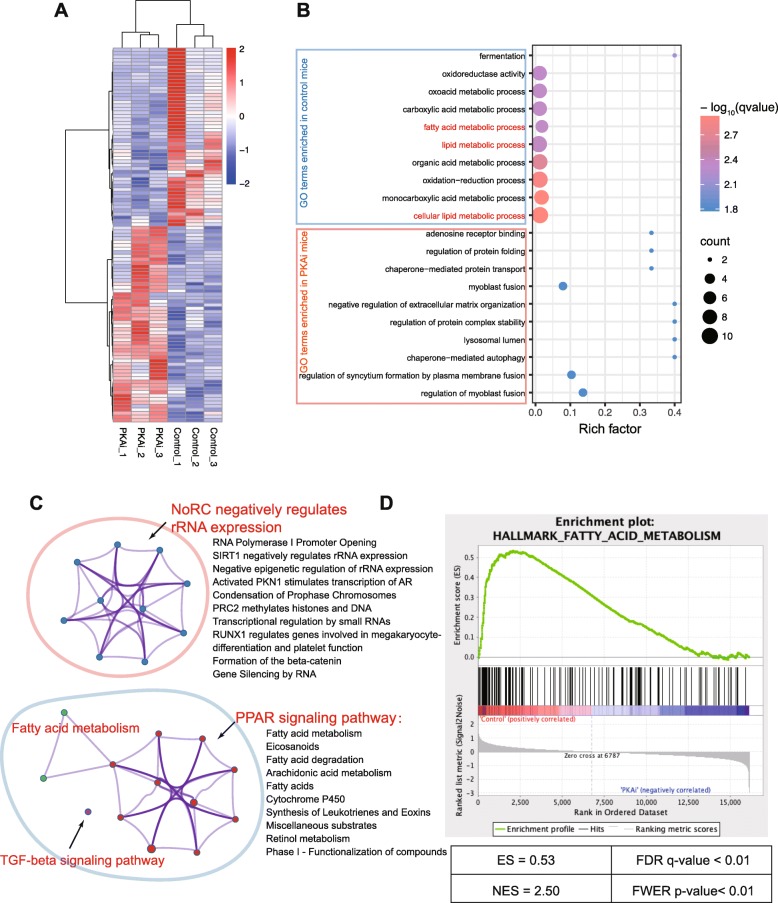
Transcriptome analysis of PKAi-GFP mice at baseline. Total mRNA was isolated from the livers of 3 PKAi and 3 control mice of 16-week old fed with chow diet, and then used to construct library for RNA sequencing. **a** The heatmap of differentially expressed genes (DEGs) of PKAi and control mice. **b** The top 10 enriched Gene ontology (GO) terms (ranked by q-value, i.e. a modified Fisher exact *P*-value) of the DEGs. Rich factor indicates the ratio of input gene number to background gene number of each term. **c** Clustered pathways of the DEGs based on KEGG and Reactome database. Each node represents a pathway, and the nodes in one color represent a cluster of pathways. The nodes in red circle were the enriched pathways of up-regulated DEGs, and the nodes in blue circle were the enriched pathways of down-regulated DEGs. The thickness of lines between nodes represents the kappa score of overlapping genes of the linking nodes, calculated by Metascape online tool. **d** The top 1 term (ranked by false discovery rate (FDR) q-value) in GSEA enrichment of Control versus PKAi mice, running with the hallmark gene sets of Molecular Signatures Database (MSigDB). The top portion of the plot shows the enrichment scores (ES) for each gene, whereas the bottom portion of the plot shows the value of the ranking metric moving down the list of ranked genes. NES: normalized enrichment score; FWER *p*-value: family wise-error rate. (*N* = 3)

PKA plays an important role in regulating lipid and glycogen metabolism in the liver [37]. We first performed the next generation RNA-seq to compare the transcriptomes of the livers of PKAi with those of control mice in order to find out the major functions and pathways affected by PKA inhibition in liver. One hundred and seven DEGs (51 down-regulated, and 56 up-regulated in PKAi mice) were screened out by analyzing the RNA-seq data. The heatmap in Fig. 2a showed the hierarchical clustering of these DEGs, which distinguished the two groups very well. Interestingly, among these DEGs, fgf21 was the most differentially expressed gene, down-regulated in PKAi mice with a log (fold-change) of 4.43 (Additional file 5). Fgf21 is a well-known protective factor in attenuating NAFLD by promoting free fatty acid oxidation [38]. GO enrichment analysis found 42 GO terms significantly enriched in up-regulated DEGs and 35 GO terms significantly enriched in down-regulated DEGs (q-value < 0.05) in PKAi mice. The top 10 GO terms, ranked by q-value, were shown in Fig. 2b. Notably, lots of metabolic-associated GO terms (such as “lipid metabolic process”, “fatty acid metabolic process” and “cellular metabolic process”) were in these DEGs. In PKAi liver, there was increased expression of genes involved in protein stability. Furthermore, the pathway enrichment of the DEGs based on KEGG and Reactome database showed that the “PPAR signaling pathway”, was the most significantly enriched pathway in PKAi mice (with both the highest rich factor of 0.188 and the lowest q-value of 6.3 × 10^− 6^) (Fig. 2c). PPARα, an isoform of PPARs family predominantly expressed in the liver, is a key transcriptional factor in promoting lipid metabolism [39]. Its significant enrichment in the down-regulated DEGs suggested that PKAi mice might had the tendency of less fatty acid degradation in the liver. Finally, we performed GSEA with all the genes detected in the RNA-seq to sensitively predict the most significant marker of the PKAi mice. As a result of the GSEA, the top 1 hallmark, ranked by FDR, is the “FATTY ACID METABOLISM” (Fig. 2d).

Together, we compared the transcriptomes of the liver in PKAi and control mice, indicating that PKAi mainly regulates the expression of genes involved in lipid metabolism.

### Hepatic-PKA inhibition had no effect on liver lipid accumulation under chow diet

**Fig. 3:**
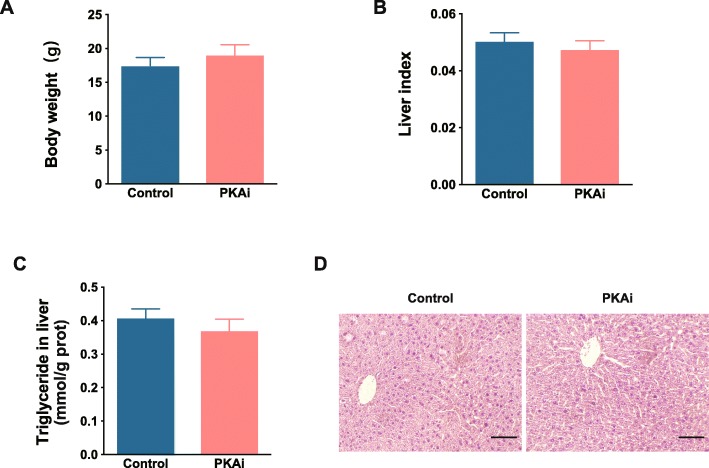
Hepatic-PKA inhibition had no significant effect on liver lipid accumulation. Mice of 16-week old fed with chow diet (CD) were used. **a** The body weight of PKAi and control mice. **b** The ratio of liver weight to body weight of PKAi and control mice. **c** The concentration of triglyceride of the livers. **d**. The representative images of H&E staining of liver tissue. Scale bar: 50 μm. (*N* = 6)

The transcriptome analysis suggested a tendency of lipid accumulation in PKAi liver. Thus, we measured their body weight, liver index (ratio of liver weight to body weight) and the triglyceride content in liver tissue. However, 16-week old PKAi mice did not show any significant difference in food consumption (Additional file 6), body weight (Fig. 3a), liver index (Fig. 3b), nor triglyceride content of the liver (Fig. 3c) compared to the control mice. Further H&E staining of liver either did not show any significant pathological difference between the two groups (Fig. 3d).

### Hepatic-PKA inhibition exacerbated HFD induced lipid accumulation in liver

**Fig. 4:**
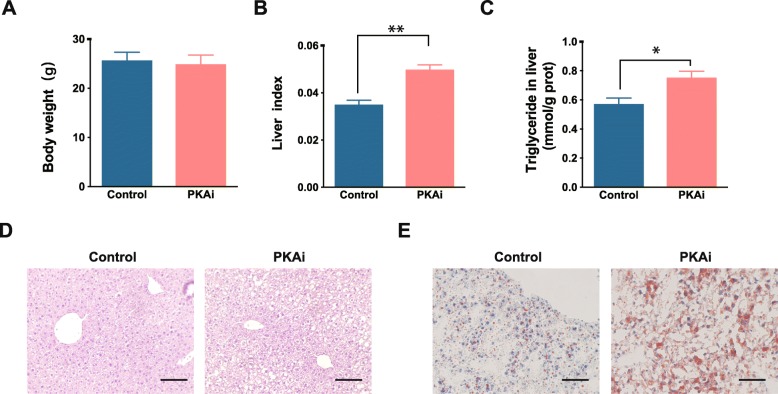
Hepatic-PKA inhibition exacerbated HFD induced lipid accumulation in liver on an early stage. Mice were fed with high fat diet (HFD) from 8-week old for 2 months. **a** The body weight of PKAi and control mice. **b** The ratio of liver weight to body weight of PKAi and control mice. **c** The concentration of triglyceride of the livers. **d-e** The representative images of H&E staining (**d**) and oil red staining (**e**) of liver tissue. Scale bar: 50 μm. *p < 0.05, **p < 0.01 vs control by t-test. (*N* = 6)

As PKAi changes the gene expression involved in lipid metabolism, we further tested lipid metabolism in the liver under metabolic stress in PKAi mice. We fed the PKAi and control mice with HFD for 2 months. Although there was no difference in the food consumption (Additional file 6) and the body weight of PKAi mice compared to control mice (Fig. 4a), the liver weight and the liver index was increased more significantly in PKAi mice (Fig. 4b), which might be attributed to more triglyceride accumulation and hepatocyte enlargement in the liver of PKAi mice (Fig. 4c-e). The accumulation of triglyceride was confirmed by severe hepatocyte vacuolation (Fig. 4d) and increased oil red O staining (Fig. 4e) in the liver of PKAi mice. Together, the inhibition of PKA activity in vivo aggravated the lipid accumulation in the liver upon HFD stimulation.

### HFD feeding differentially altered transcriptomes in control and hepatic-PKAi mice

Given PKAi mice showed greater lipid accumulation in the liver than the control mice under HFD treatment, next we sought to have a deeper insight of the mechanism and consequences behind this phenomenon. We compared the transcriptome alterations in the liver of control mice or PKAi mice with HFD versus with CD respectively, which enables us to illustrate the different effects of HFD on control mice and PKAi mice.

**Fig. 5:**
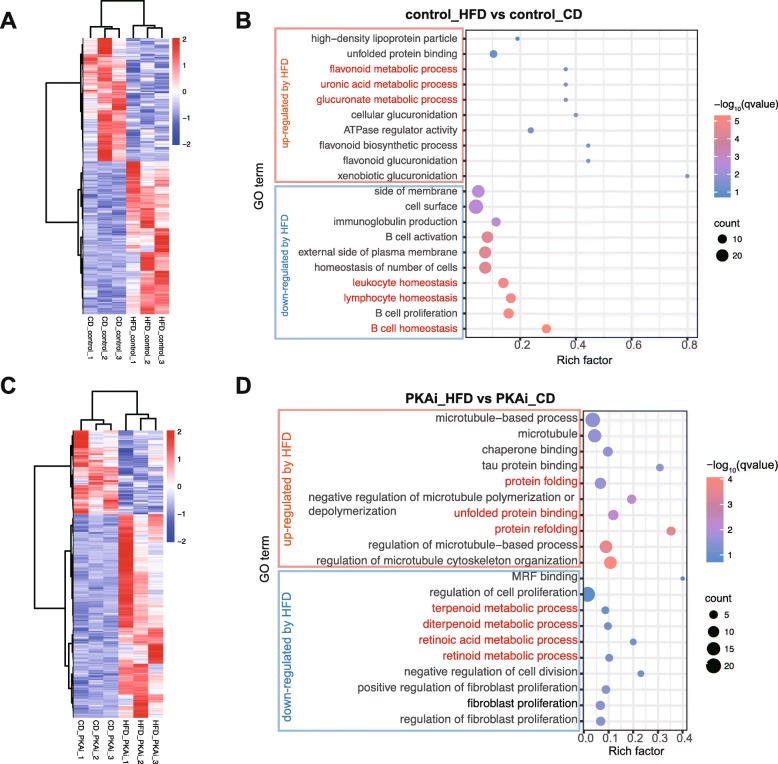
HFD effects on transcriptome of PKAi and control mice. Mice were fed with HFD or CD from 8-week old for 2 months. Liver tissues were harvested to perform the RNA sequencing. **a-b** The heatmap (**a**) and top 10 enriched GO terms (**b**) of DEGs in the liver tissue of control mice fed with CD vs. HFD. **c-d** The heatmap (**c**) and top 10 enriched GO terms (**d**) of DEGs in the liver tissue of PKAi mice fed with CD v.s. HFD. GO terms were ranked by qvalue, the modified Fisher exact P-value. Rich factor indicates the ratio of input gene number to background gene number of each term. (*N* = 3)

First, we explored the effects of HFD on the transcriptomes of the liver tissues of control and PKAi mice respectively. In the control mice, 397 genes were up-regulated by HFD and 321 genes were down-regulated by HFD (Fig. 5a). GO enrichment analysis (Fig. 5b) showed that inf control mice HFD feeding increased the expression of genes enriched in high-density lipoprotein particle, protein folding, flavonoid metabolism, as well as glucose metabolism (uronic acid, glucuronate metabolism), while the down-regulated genes were enriched in the inflammation/immunity homeostasis. On the other hand, in PKAi mice, 332 genes were up-regulated while 137 genes were down-regulated by HFD (Fig. 5c). Interestingly, the up-regulated genes were enriched in protein folding GO terms, such as “protein folding”, “unfolded protein binding”, suggesting that severe endoplasmic reticulum (ER) stress was induced by HFD in PKAi mice. However, the down-regulated genes were mainly enriched in cell proliferation and some metabolic processes (terpenoid, diterpenoid, retinoic acid and retinoid). (Fig. 5d).

**Fig. 6:**
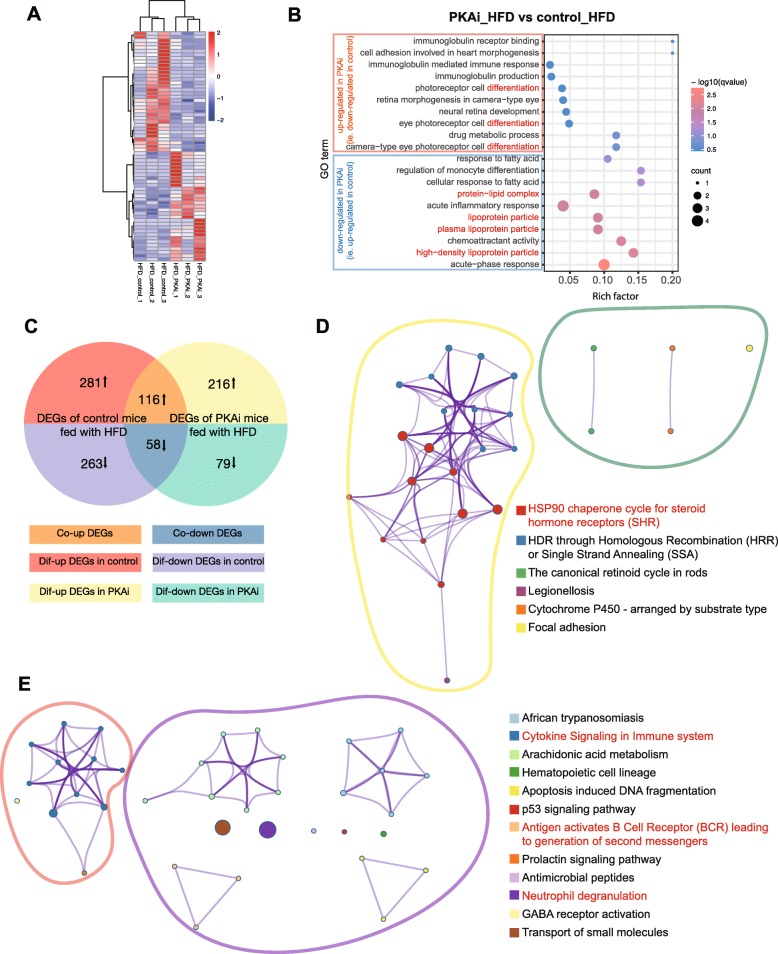
The unique transcriptome alteration of PKAi and control mice induced by HFD. Mice were fed with HFD or CD from 8-week old for 2 months. Liver tissues were harvested to perform the RNA sequencing. **a-b** The heatmap (**a**) and top 10 enriched GO terms (**b**) of DEGs in the liver tissue of control mice vs. PKAi mice with HFD feeding. **c** Venn diagram showing the co-altered or differentially-altered DEGs in PKAi and control mice: orange and blue areas represent co-altered genes, either up- (orange) or down-regulated (blue) on the stimulation of HFD; red and purple areas represent differentially-altered genes in control mice, either up- (red) or down-regulated (purple) on the stimulation of HFD; yellow and green areas represent differentially-altered genes in PKAi mice, either up- (yellow) or down-regulated (green) after the stimulation of HFD. **d-e** Enriched pathways of differentially-altered genes in PKAi mice (**d**) and control mice (**e**). Each node represents a pathway, and the nodes in one color represent a cluster of pathways. The nodes in different-colored circles represent the enriched pathways of DEGs of the same color displayed in **c**. The thickness of lines between nodes represents the kappa score of overlapping genes of the linking nodes, calculated by Metascape online tool. (*N* = 3)

Then, we compared the transcriptome of liver tissue of control mice and PKAi mice both with HFD treatment. We found 34 genes were expressed less and 31 gene were expressed more in HFD-induced PKAi mice compared to HFD-induced control mice (Fig. 6a). Further GO analysis of these DEGs (Fig. 6b) showed that genes with lower expression in PKAi_HFD mice (i.e. higher in control mice) were mainly enriched in GO terms with “lipoproteins”, which are critical to transport fatty acids, TG and cholesterol out of hepatocytes. This may explain why there was more TG accumulation in PKAi_HFD mice liver. On the other hand, DEGs with higher expression level in PKAi_HFD mice seemed to be mostly enriched in cell differentiation and immune response.

Finally, we compared the changing directions of DEGs induced by HFD in control and in PKAi mice, which might provide more specific clues to illustrate the different responses to HFD between the two types of mice. By merging the 2 sets of DEGs of the control and PKAi mice effected by HFD (Fig. 6c), we found that 174 genes (termed “co-up/down DEGs” as shown in Fig. 6c) had the same changing direction in both groups (116 up-regulated by HFD, 58 down-regulated by HFD). However, 544 and 295 DEGs (termed “dif-up/down DEGs” as shown in Fig. 6c) changed in different directions in control mice (281 up-regulated by HFD, 263 down-regulated by HFD) and PKAi mice (216 up-regulated, 79 down-regulated), respectively, which could reflect the different responses to HFD feeding between the two types of mice at the mRNA level. Based on the Reactome and KEGG database, we did pathway enrichment, finding that the unique DEGs in PKAi mice were mostly enriched in “HSP90 chaperone cycle for steroid hormone receptors (SHR)” and “HDR through Homologous Recombination (HRR) or Single Strand Annealing (SSA)”, which were closely related to cellular response to stress and DNA repair responding to ER stress (Fig. 6d). On the other hand, the unique DEGs in control mice were mostly enriched in pathways related to immune system, such as “Cytokine Signaling in Immune system”, “Antigen activates B Cell Receptor (BCR) leading to generation of second messengers” and “Neutrophil degranulation” (Fig. 6e). These results suggested that HFD caused immune homeostasis alterations in the liver of control mice while activated direct response to stress and DNA repair mechanism in PKAi mice.

## Discussion

In the present study, for the first time we demonstrated that hepatic-PKA inhibition could result in lipid accumulation in the liver, a hallmark of NAFLD [4]. PKAi in hepatocytes not only altered the transcription of genes involved in metabolism of fatty acids and lipids, but also altered the transcriptome response to HFD in the liver. This report provides the first direct evidence of a potential role of PKA in NAFLD development and shed lights on the mechanisms of PKA regulated lipid metabolism in liver physiology and pathology.

As there is no specific PKA inhibitor that can achieve liver-specific PKA inhibition and it is very challenging to make genetic loss-of-function mouse by knocking out all PKA subunits, it has been long sought to determine the role of PKA in liver function in vivo. In this the research area, G. Stan McKnight has constructed many mouse models with single PKA subunit knockout. Zheng et al. reported that disruption of PKA RIIβ subunit in mice adipose tissue leads to a lean phenotype, increased nocturnal locomotor activity, and activation of brown adipose tissue [40]. However, the activity of PKA has not been determined in this study, which might be compensated by RIα at the molecular level. London et al. constructed a ubiquitously ablated PKA RIIα mouse model with a phenotype of resistance to diet-induced obesity, glucose intolerance, and hepatic steatosis [41]. But the global deficiency of RIIα caused different activities of PKA in some key metabolic organs: cAMP-stimulated PKA activity was decreased in liver but increased in gonadal adipose tissue as reported, which makes it hard to draw the conclusion that the in vivo phenotype is due to the activation or the inhibition of PKA. On the other hand, studies on catalytic subunits also remain ambiguous. Linda et al. constructed a catalytic Cβ global knockout mice, which also protected mice from diet-induced obesity, steatosis, dyslipoproteinemia, and insulin resistance, without any differences in caloric intake or locomotor activity [42]. Theoretically Cβ knockout will lead to a low activity of PKA, which might lead to a decrease of lipolysis as suggested in vitro studies and our results, but a lean phenotype was observed in vivo. This could be due to the compensatory changes of the expression/activity of other PKA catalytic subunits. Therefore, it is difficult to elucidate the role of PKA in liver physiology and pathology. Here we inhibited PKA activity in a hepatic-specific, controlled by *Alb* promoter with a double transgenic mouse system to overexpress a PKA inhibiting peptide (PKAi-GFP). In our mouse model, the overexpression of PKAi-GFP successfully inhibited PKA activity in the liver, thus allowing us for the precise and comprehensive study of the roles of PKA in liver tissue. In future studies, it is possible to study the effects of different degrees of PKA inhibitions on the development of NAFLD by screening out transgenic mouse strains with different abundance of PKAi expression, and even achieve the functional PKA ablation model through extremely high expression as reported recently [43].

In the present study, the inhibition of PKA activity in the liver showed a normal liver morphology when fed with chow diet and a rapid lipid accumulation in the liver after HFD induction. Our transcriptome study showed considerable differences in the transcriptome of PKAi mice with normal chow feeding, and these differences are largely enriched in lipid metabolism. However, changes in the RNA level appear to be insufficient to cause overt disease phenotype when fed with a control diet. The phenotype could be too subtle or needs more time to develop, such as in ageing mice, which warrants further study. Using HFD as an external stimulus, PKAi mice rapidly developed lipid accumulation in the liver. The development of TG accumulation could be due to multiple reasons: first, our transcriptome analysis suggested that reduced lipid metabolism in hepatocytes of PKAi liver; when fed with HFD chow, PKAi mice had less lipoprotein expression. Lipoproteins play a key role in the transportation of lipids from the liver to peripheral tissues. In this study, some genes related to lipoproteins were up-regulated in control mice when fed with HFD, but were not induced by HFD in PKAi mice, which might account for the less TG accumulation in the liver of control mice. In addition, PKA directly regulates lipase activity in the liver and other metabolic tissues like adipose tissue via phosphorylating these enzymes as reported in the previous in vitro studies [44, 45]. These aspects were not explored in this study to make the study more focused though they need to be studied in the future.

Excessive import or diminished export or oxidation of free fatty acids is the initial step of NAFLD, which activates the immune system to protect the hepatic cells from damage [3]. Once hepatic immune cell is overactivated, inflammation will take place and lead to the NASH. This explains why the immune response genes showed a significant enrichment in control mice after HFD treatment for 8 weeks. In contrast, DEGs in PKAi mice mainly enriched in protective cellular responses to stimuli, including “HSP90 chaperone cycle for steroid hormone receptors (SHR)” and “HDR through Homologous Recombination (HRR) or Single Strand Annealing (SSA)”. PKA can be activated by various of hormones, such as catecholamines [46], parathyroid hormone [47], and glucagon [48], and then drives lipolysis. Therefore, inhibition of PKA in the liver may alter the reactivity of the hormones induced response to stimuli. However, one limitation of our study is that we only explored a broad transcriptional alteration by the inhibition of PKA in the liver and provided predictive possible mechanisms, which invokes more experiment-based studies in the future.

In our study, 8 weeks of HFD was sufficient to induce hepatic steatosis in PKAi mice, which is 4–8 weeks shorter than the time needed for HFD to induce NAFLD in control mice in previous reports [49]. This finding suggests that inhibition of PKA promotes lipid accumulation in the liver, and our PKAi mice fed with HFD can be potentially used as an early stage NAFLD mouse model. It further suggests that reduced PKA activity could be a contributing factor for the development of NAFLD and activating PKA might be a novel strategy to combat the development of NAFLD.

## Conclusions

Together, our data demonstrated that hepatic PKA inhibition alters the lipid metabolism pathways and is sufficient to induce rapid lipid accumulation under HFD treatment.

## Supplementary information

**Additional file 1.** The formulation of CD and HFD used in the study was demonstrated in the table

**Additional file 2.** The detail information of the antibodies used in Western blot experiments are listed in the table

**Additional file 3.** The sequence information of the primers used in qRT-PCR are listed in the table

**Additional file 4 **Total RNA was extracted from different tissues of PKAi-GFP mice and qRT-PCR was used to test the PKAi-GFP mRNA expression. **p < 0.01 vs liver tissue by one-way ANOVA with with the Tukey–Kramer post hoc test. (*N* = 3)

**Additional file 5. **The gene symbols, fold change and adjusted-*p* values of the DEGs of PKAi and control mice are listed in the table

**Additional file 6. **Mice were fed with CD or HFD from 8-week old for 2 months. Twenty-four-hour of food consumption of each group was measured weekly. (*N* = 6)

## Data Availability

All data generated or analyzed during this study are included in this published article or are available from the corresponding author on reasonable request.

## Abbreviations

cAMP: cyclic adenosine monophosphate
CD: chow diet
CREB: cAMP response element binding protein
DEG: differentially expressed gene
FDR: false discovery rate
GO: Gene Ontology
GSEA: gene set enrichment analysis
HFD: high-fat diet
HSL: hormone sensitive lipase
KEGG: Kyoto encyclopedia of genes and genomes
MSigDB: Molecular Signatures Database
NAFLD: nonalcoholic fatty liver disease
NASH: nonalcoholic steatohepatitis
PKA: protein kinase A
PKB: protein kinase B
PPARα: peroxisome proliferators-activated receptors alpha
RNF-20: ring finger protein-20
RPKM: reads per kilobase per million mapped reads
SRA: Sequence Read Archive
SREBP-1c: sterol regulatory element binding protein-1c
