# Supplementary material for: Hepatic PKA inhibition accelerates the lipid accumulation in liver
Source: Nutr Metab (Lond). 2019 Oct 11;16:69. doi: 10.1186/s12986-019-0400-5 (PMC6788098; doi:10.1186/s12986-019-0400-5)
Supplement: Supplementary file 1 — Additional file 1. The formulation of CD and HFD used in the study was demonstrated in the table [file 12986_2019_400_MOESM1_ESM.docx]

**Formula of HFD (D12451, Research Diets, US)**

| Class description | Ingredient | Grams |
| --- | --- | --- |
| Protein | Casein, Lactic, 30 Mesh | 200.00 g |
| Protein | Cystine, L | 3.00 g |
| Carbohydrate | Sucrose, Fine Granulated | 176.80 g |
| Carbohydrate | Lodex 10 | 100.00 g |
| Carbohydrate | Starch, Corn | 72.80 g |
| Fiber | Solka Floc, FCC200 | 50.00 g |
| Fat | Lard | 177.50 g |
| Fat | Soybean Oil, USP | 25.00 g |
| Mineral | S10026B | 50.00 g |
| Vitamin | Choline Bitartrate | 2.00 g |
| Vitamin | V10001C | 1.00 g |
| Dye | Dye, Red FD&C #40, Alum. Lake 35-42% | 0.05 g |
|  | Total: | 858.15 g |

**Formula of CD (D12450B, Research Diets, US)**

| Class description | Ingredient | Grams |
| --- | --- | --- |
| Protein | Casein, Lactic, 30 Mesh | 200.00 g |
| Protein | Cystine, L | 3.00 g |
| Carbohydrate | Sucrose, Fine Granulated | 354.00 g |
| Carbohydrate | Starch, Corn | 315.00 g |
| Carbohydrate | Lodex 10 | 35.00 g |
| Fiber | Solka Floc, FCC200 | 50.00 g |
| Fat | Soybean Oil, USP | 25.00 g |
| Fat | Lard | 20.00 g |
| Mineral | S10026B | 50.00 g |
| Vitamin | Choline Bitartrate | 2.00 g |
| Vitamin | V10001C | 1.00 g |
| Dye | Dye, Yellow FD&C #5, Alum. Lake 35-42% | 0.05 g |
|  | Total: | 1055.05 g |
