# Supplementary material for: Hepatic PKA inhibition accelerates the lipid accumulation in liver
Source: Nutr Metab (Lond). 2019 Oct 11;16:69. doi: 10.1186/s12986-019-0400-5 (PMC6788098; doi:10.1186/s12986-019-0400-5)
Supplement: Supplementary file 2 — Additional file 2. The detail information of the antibodies used in Western blot experiments are listed in the table [file 12986_2019_400_MOESM2_ESM.docx]

**Antibodies used in the western blot experiment**

| Antibody | Dilution | Supplier | Product number |
| --- | --- | --- | --- |
| GFP | 1:1000 | Abcam, UK | ab290 |
| β-actin | 1:1000 | Cell Signaling Technology, US | #4970 |
| Anti-rabbit IgG | 1:5000 | Cell Signaling Technology, US | #7074 |
