# Supplementary material for: Hepatic PKA inhibition accelerates the lipid accumulation in liver
Source: Nutr Metab (Lond). 2019 Oct 11;16:69. doi: 10.1186/s12986-019-0400-5 (PMC6788098; doi:10.1186/s12986-019-0400-5)
Supplement: Supplementary file 3 — Additional file 3. The sequence information of the primers used in qRT-PCR are listed in the table [file 12986_2019_400_MOESM3_ESM.docx]

**Sequence information of primers used in qRT-PCR**

| Target gene | Primer sequence |
| --- | --- |
| PKAi-GFP | F: 5’- CATGTCTGGATCGATCCCCG -3’  R: 5’- CCCTTGCTCCATACCACCCC -3’ |
| β-actin | F: 5’- CTACAATGAGCTGCGTGTGG-3’  R: 5’- AAGGAAGGCTGGAAGAGTGC-3’ |
