# Supplementary figures and images for: Hepatic PKA inhibition accelerates the lipid accumulation in liver
Source: Nutr Metab (Lond). 2019 Oct 11;16:69. doi: 10.1186/s12986-019-0400-5 (PMC6788098; doi:10.1186/s12986-019-0400-5)

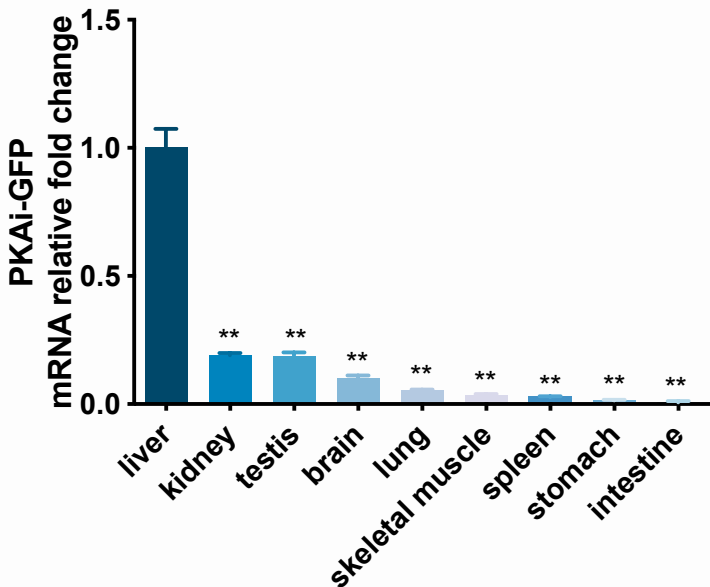

Supplement: Supplementary file 4 — Additional file 4 Total RNA was extracted from different tissues of PKAi-GFP mice and qRT-PCR was used to test the PKAi-GFP mRNA expression. **p < 0.01 vs liver tissue by one-way ANOVA with with the Tukey–Kramer post hoc test. (N = 3) [file 12986_2019_400_MOESM4_ESM.pdf]

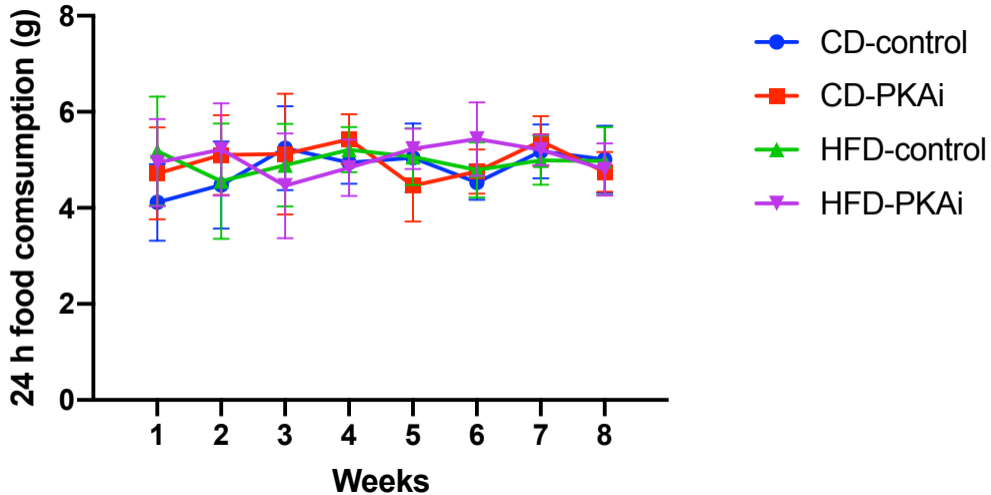

Supplement: Supplementary file 6 — Additional file 6. Mice were fed with CD or HFD from 8-week old for 2 months. Twenty-four-hour of food consumption of each group was measured weekly. (N = 6) [file 12986_2019_400_MOESM6_ESM.pdf]
